# Complete Genome Sequence and Comparative Genomics of a Novel Myxobacterium *Myxococcus hansupus*

**DOI:** 10.1371/journal.pone.0148593

**Published:** 2016-02-22

**Authors:** Gaurav Sharma, Tarun Narwani, Srikrishna Subramanian

**Affiliations:** CSIR-Institute of Microbial Technology, Sector-39A, Chandigarh, India; Tianjin University, CHINA

## Abstract

Myxobacteria, a group of Gram-negative aerobes, belong to the class δ-proteobacteria and order Myxococcales. Unlike anaerobic δ-proteobacteria, they exhibit several unusual physiogenomic properties like gliding motility, desiccation-resistant myxospores and large genomes with high coding density. Here we report a 9.5 Mbp complete genome of *Myxococcus hansupus* that encodes 7,753 proteins. Phylogenomic and genome-genome distance based analysis suggest that *Myxococcus hansupus* is a novel member of the genus *Myxococcus*. Comparative genome analysis with other members of the genus *Myxococcus* was performed to explore their genome diversity. The variation in number of unique proteins observed across different species is suggestive of diversity at the genus level while the overrepresentation of several Pfam families indicates the extent and mode of genome expansion as compared to non-Myxococcales δ-proteobacteria.

## Introduction

Myxobacteria are Gram-negative δ-proteobacteria [1, 2] which are mostly aerobic with some notable exception such as *Anaeromyxobacter* [3]. A peculiar trait of Myxobacteria is their social communication within swarms [2] wherein numerous cell-cell interactions define some of their physiological attributes such as gliding motility [4], fruiting body formation [5], biofilm production, [6] and hunting prey characteristics [7]. Myxobacteria display gliding movement like cyanobacteria and flexibacteria, however, the process is more distinct [8] exhibiting two different types of motilities viz., adventurous and social. Adventurous motility (A) is attributed to a single cell while coordinated movement by a swarm is termed as social motility (S) [9]. Under starvation conditions, Myxobacteria form complex fruiting bodies composed of dormant myxospores, analogous to stalk formation in higher-order fungi [10, 11]. Owing to their complex life cycle, Myxobacteria contain many proteins involved in signal transduction pathways and transcriptional regulation [2]. These proteins help in regulating cell-cell communication and coordinate social motility and fruiting body formation. Besides these unique physiological properties, the relatively large genome size (4.5–15 Mbp) is a characteristic feature of order Myxococcales that distinguish it from other δ-proteobacteria (typically 2–7 Mbp) [5, 12, 13]. The smallest member of the order Myxococcales is *Vulgatibacter incomptus* DSM 27710 with a genome size of 4.35 Mbp (CP012332.1) followed by *Anaeromyxobacter* with a genome size of ~5 Mbp, which is comparable in size to other non-Myxococcales δ-proteobacteria [3]. However, the myxobacterium *Sorangium cellulosum* So0157-2 (14.78 Mbp) [12] is one of the largest genomes among the bacterial clade known till date. The expansion of genome size in Myxococcales is reported to be widespread in all constituent families like; Myxococcaceae, Cystobacteraceae, Kofleriaceae and Polyangiaceae [5]. Expansion of a genome indicates increased complexity, influenced by environmental factors and occurrence of genetic events such as duplication and integration of foreign genes via horizontal gene transfer [14]. A large number of duplicated proteins found in Myxobacterial genomes has been suggested to help it adapt to diverse habitats and help in its complex life cycle [15].

Here we report a novel Myxobacterial genome which was found growing as a contaminant in a culture plate of *Chondromyces apiculatus* DSM436 procured from the DSMZ culture collection. We have assembled the complete genome of *Myxococcus hansupus* (named after Dr. Hans Reichenbach and herein referred to as *M*. *hansupus* or *Mh*) and performed its comparative genome analysis with all available genomes in the genus *Myxococcus* viz., *M*. *xanthus DK1622*, *M*. *fulvus HW-1*, *M*. *stipitatus*, *M*. *xanthus DZ2* and *M*. *xanthus DZF1* [15–19]. Analysis of these genomes was carried out to help understand the extent of conservation and variability of proteomes in these closely related organisms.

## Material and Methods

### Culturing and DNA isolation of *M*. *hansupus*

**Fig 1:**
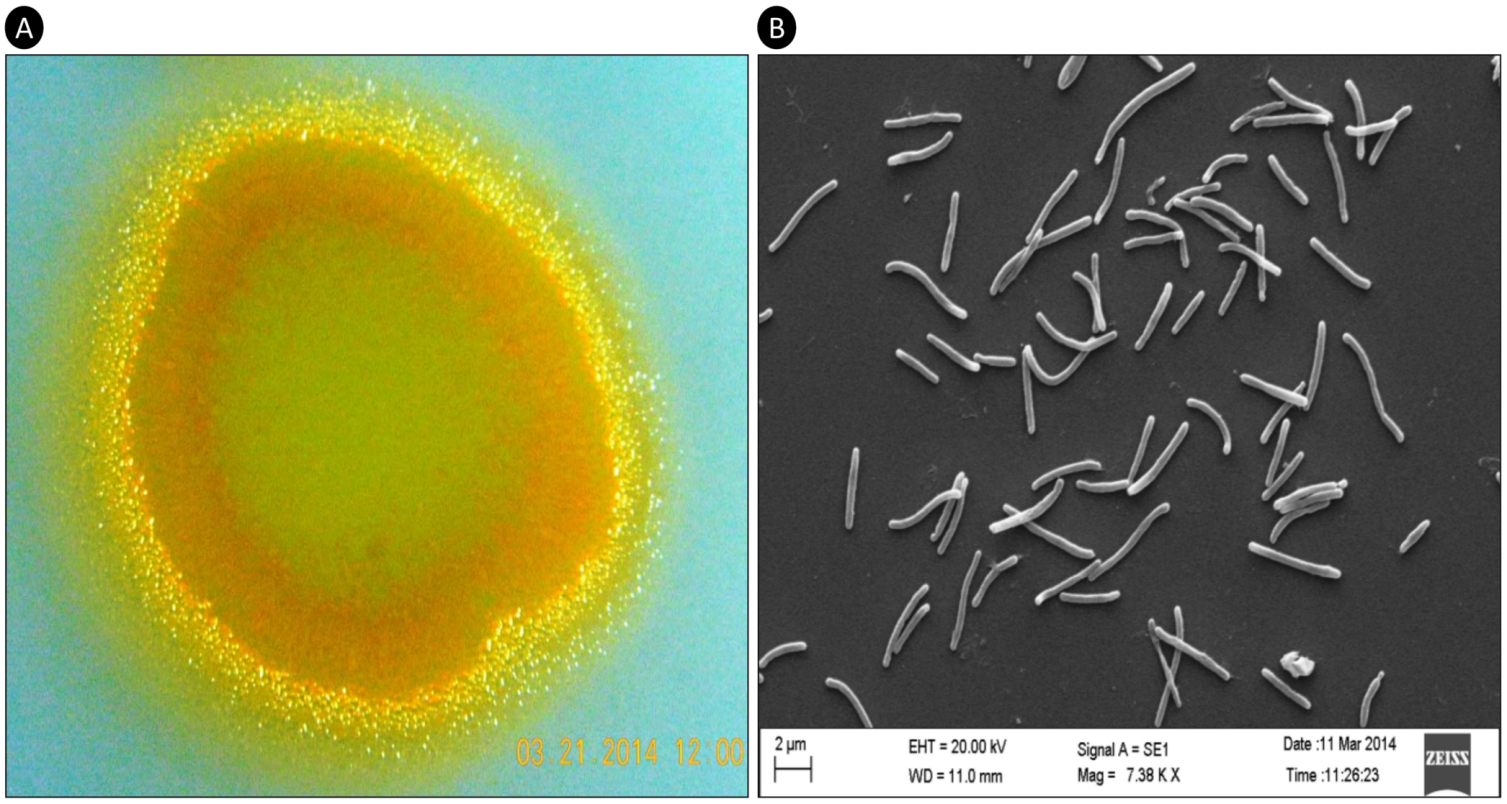
Colony and cellular morphology. **A.** Colony morphology of *M*. *hansupus* swarm with 1x magnification; **B.** Cellular morphology as visualized with Scanning Electron Microscopy (SEM) with 7,380x magnification.

*M*. *hansupus* was purified from a contaminant on the culture plate of *Chondromyces apiculatus*, procured from Deutsche Sammlung von Mikroorganismen und Zellkulturen (DSMZ) culture collection as strain number DSM-436. It was grown on VY/2 agar and SP agar medium plates and is reddish yellow in color (Fig 1A). The swarms were soft and slimy, evenly spread as a film on the agar surface, unlike the *Chondromyces* whose swarms imprint shallow depressions and ridges on the agar [20]. Under scanning electron microscope they look rod-shaped (Fig 1B). Whole genomic DNA was isolated from the pure culture using ZR Fungal/Bacterial DNA MicroPrep^™^. 16S rRNA sequencing of the isolated DNA was performed using universal bacterial primers at our in-house Sanger sequencing facility. The strain was named *M*. *hansupus* and is maintained in our laboratory as an actively growing culture.

### Whole genome sequencing and assembly of *M*. *hansupus*

Sequencing was performed on a Pacific Biosciences RSII instrument at the Genome Quebec Innovation Center, McGill University, Montréal (Québec), Canada. SMRTbell library was constructed with 10 μg whole genomic DNA using a 20 kb Template Preparation method and BluePippin^™^ Size Selection. The library was then loaded onto two single-molecule real-time (SMRT) cells and sequenced using P6 polymerase and C4 chemistry (P6C4) with 180-minute movie times. Sequencing yielded a total of 145,073 reads with a mean read length of 10,730 bp and 1,556,757,303 bp with an estimated coverage of 138×. *De novo* assembly was carried out using the hierarchical genome assembly process (HGAP) protocol from SMRT Analysis v2.0, including consensus polishing with Quiver [21]. Gene prediction and functional annotation were performed by Rapid Annotation using Subsystem Technology (RAST) [22]. RNAmmer 1.2 and tRNAscan-SE-1.23 were used to predict rRNA and tRNA genes [23, 24]. The complete genome was used as a reference to determine the putative methylome of *M*. *hansupus* genome using base modifications and enriched motifs identification protocol of the SMRT portal.

### Data source for comparative genome analysis

Besides the complete genome of *M*. *hansupus* (*Mh*; CP012109), genome sequences of *M*. *xanthus DK1622* (*MxDK1622*; NC_008095.1) [25]; *M*. *fulvus HW-1* (*Mf*; NC_015711.1) [26]; *M*. *stipitatus* [*Ms*; NC_020126.1] [16]; *M*. *xanthus DZ2* (*MxDZ2*; AKYI00000000) [18] and *M*. *xanthus DZF1* (*MxDZF1*; AOBT00000000) [17] were obtained from NCBI for this study. *MxDK1622*, *Mf*, and *Ms* are complete genomes while *MxDZF1* and *MxDZ2* are draft assemblies. For all these genomes, gene prediction and functional annotation were done using Rapid Annotation using Subsystem Technology (RAST). We also analyzed the replication origin in *M*. *hansupus* and compared it with those identified in other *Myxococcus* genomes. Complete genomes of *M*. *hansupus* and other genus *Myxococcus* members were subjected to BLASTn against oriC sequences available at the DoriC database [27, 28].

### Gene identification and reannotation of myxobacterial genomes

In order to have similar annotations for comparative genomics, and to identify annotation inconsistencies, we subjected all the aforementioned genomes to different gene calling and annotation protocols. Various annotation pipelines like RAST [22], GLIMMER [29], xBASE [30] were used in this study using a minimum gene length of 100 bp. Annotated protein-sets from all pipelines were mapped to each other along with the original dataset available in NCBI using BLASTp [E-value cutoff of 1e^-5^]. For all genome and pipeline combinations, percentage mapping within each annotation combination was calculated.

### Phylogenetic analysis of *M*. *hansupus* using 16S rRNA and housekeeping proteins

16S rRNA sequences from the genus *Myxococcus* were extracted from NCBI. Forty *Myxococcus* 16S rRNA sequences along with five out-group sequences (one from each of C*orrallococcus*, *Cystobacter*, *Anaeromyxobacter*, *Sorangium* and *Bdellovibrio* groups) were aligned using the ClustalW module of BIOEDIT sequence alignment tool (version 7.1.3.0) [31]. Post alignment, all the gaps were excluded and the resulting alignment was used in MEGA 6.06 [32] to generate a maximum likelihood tree [model: Tamura 3-param; bootstrap: 100]. Using the Neighbor-Joining method, initial tree(s) for the heuristic search were obtained and pairwise distance matrix was estimated using the Maximum Composite Likelihood approach. Newick notation of the tree was extracted and used as input in iTOL [33] to generate an interactive phylogenetic tree. Further, genus *Myxococcus* phylogeny was studied using conserved housekeeping genes. Twenty-eight housekeeping genes (*dnaG*, *frr*, *nusA*, *pgk*, *pyrG*, *rplC*, *rplD*, *rplE*, *rplF*, *rplK*, *rplL*, *rplM*, *rplN*, *rplP*, *rplS*, *rplT*, *rpmA*, *rpoB*, *rpsB*, *rpsC*, *rpsE*, *rpsI*, *rpsJ*, *rpsK*, *rpsM*, *rpsS*, *smpB* and *tsf*) [34] were found to be conserved in the complete and draft genomes under investigation (six *Myxococcus* genera, four neighbor genera and one non-*Myxococcales* δ-proteobacteria genus, *Bdellovibrio*). Protein sequences of these housekeeping genes were extracted from each genome and concatenated. These concatenated sequences were aligned using ClustalW module of BIOEDIT sequence alignment tool. Gaps were excluded post alignment and the resulting alignment was used as an input in MEGA 6.06 [32] to generate Maximum Likelihood tree [model: Jones-Taylor-Thornton (JTT) matrix; bootstrap: 100]. Initial tree(s) for the heuristic search were obtained by applying the Neighbor-Joining method to a matrix of pairwise distance estimated using a JTT model.

### Orthology, homology and protein clustering study

Orthology was predicted among protein datasets of the six genomes using the Reciprocal Best Hits (RBH) BLAST approach of Proteinortho [35] with an E-value cutoff of 1e^-5^, minimum query coverage of 50% and minimum identity of 35%. The program first performs an all-against-all BLASTp alignment and then defines putative orthology-pairs based on reciprocal BLAST scores. A cluster is defined by the presence of a protein in at least two genomes. NCBI BLAST+ (v 2.2.26+) was used throughout the study [36].

Homology at protein level was studied among all genomes. Protein dataset from each genome was mapped against the other using BLASTp with an E-value cutoff of 1e^-5^, minimum query coverage of 50% and minimum identity of 35%. A binary approach was followed to analyze the occurrence of each protein in different genomes. A binary map was generated based on the count of each protein’s presence/absence in various genome combinations. For clustering analysis, protein dataset from each genome was mapped against the same using BLASTp with an E-value cutoff of 1e^-5^, minimum query coverage of 50% and minimum identity of 35%. The filtered dataset for each genome was used to identify the clusters sharing all possible homologs. The mummer program from MUMmer 3.5 suite was used to generate alignment between genome pairs with a minimum alignment length cutoff of 50 bp and mummerplot was used to generate synteny plots [37].

### Pfam domain analysis and core family identification

The proteome of the six *Myxococcus* members and other order Myxococcales members were scanned against the Pfam-A v 28.0 database [38] with an E-value threshold of 1e^-5^ to identify functional domains and other known sequence motifs using hmmscan program of HMMER suite (http://hmmer.janelia.org/) [39]. The distribution of Pfam domain families among all genomes was analyzed.

## Results and Discussion

### Genome assembly and annotation

**Fig 2:**
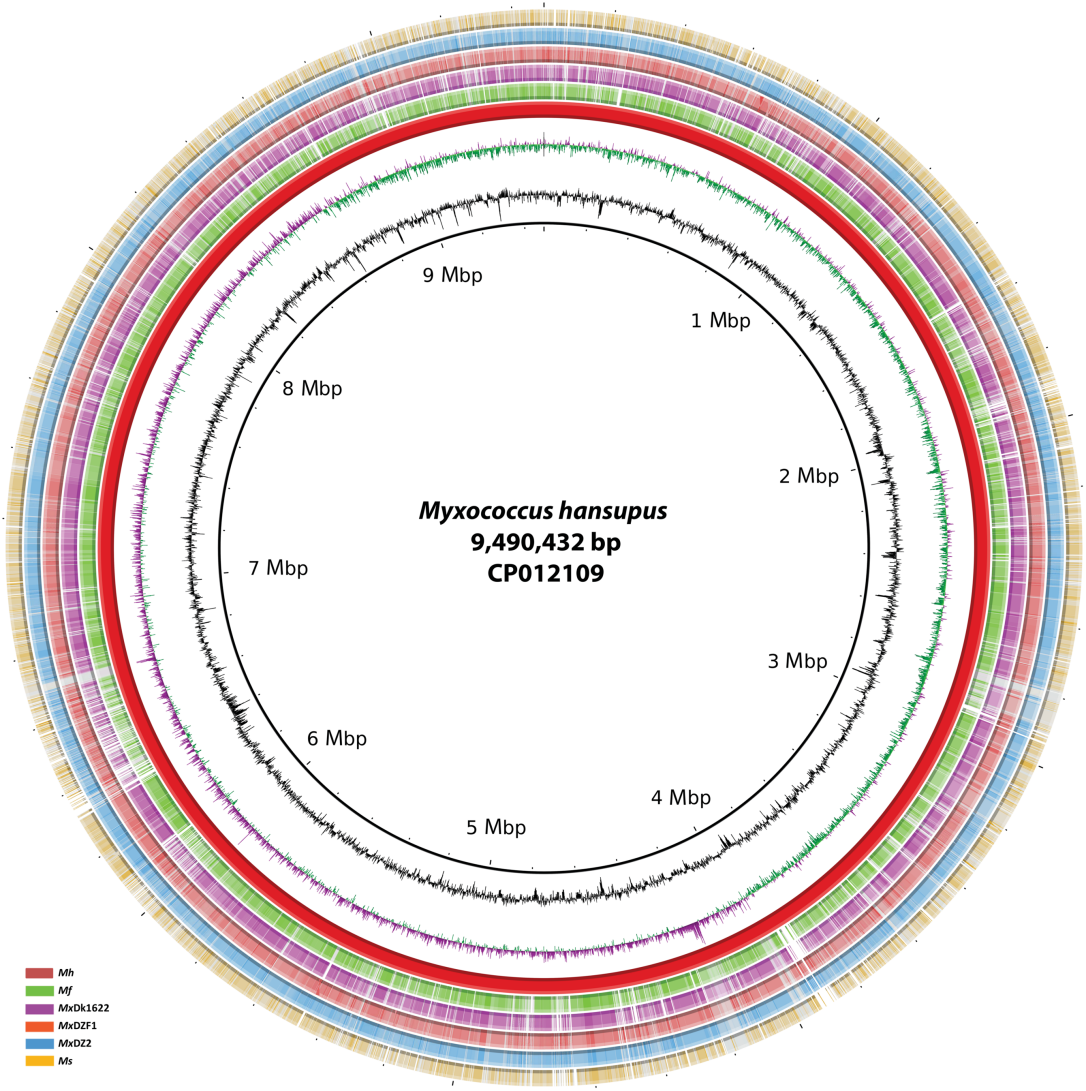
Circular representation of the *M*. *hansupus* complete genome. **Circles** (from inside to outside) **1 and 2** (GC content; black line and GC skew; magenta and green lines), **circle 3** (*M*. *hansupus*; red circle); **circle 4** (mapped *Myxococcus fulvus* HW-1 genome with *M*. *hansupus* genome; green circle); **circle 5** (mapped *Myxococcus xanthus* DK1622 genome with *M*. *hansupus* genome; purple circle); **circle 6** (mapped *Myxococcus xanthus* DZF1 genome with *M*. *hansupus* genome; Orange circle); **circle 7** (mapped *Myxococcus xanthus* DZ2 genome with *M*. *hansupus* genome; blue circle); **circle 8** (mapped *Myxococcus stipitatus* genome with *M*. *hansupus* genome; yellow circle). BRIG 0.95 was used to build the circular representation [53]. Mapping studies were done using BLASTn with an E-value cut-off 1e^-5^.

*M*. *hansupus* genome was assembled as a single chromosome of 9,490,432 nucleotides (Fig 2). The GC content is 69.2% and is comparable to other Myxobacteria [5, 15]. The RNA analysis of the genome reported four rRNA operons (5S-16S-23S) and 67 amino acyl-tRNA synthetase genes for all twenty amino acids. RAST based annotation helped identify 7,753 coding genes, out of which 4,953 proteins (63.89%) were functionally annotated while the remaining (36.11%) are hypothetical proteins. The coding density of the genome is 88.87% with an average gene length of 1088 bp. This Whole Genome Shotgun project has been deposited at DDBJ/EMBL/GenBank under the accession CP012109. The genomic features are listed in Table 1.

**Table 1:** Assembly statistics for *M*. *hansupus*.

Organism name	*Myxococcus hansupus*
Sequencing data	PacBio P6C4 chemistry sequencing
Total Reads	145,073
Number of Bases	1,556,757,303 bp
Mean Read Length	10,730 bp
Average Reference Coverage	138.05 X
Bio-Project Number	PRJNA167109
NCBI Accession number	CP012109
Genome size	9,490,432 bp
GC content	69.2%
Chromosome	1
CDS	7,753
% Coding density	88.87
CDS from (+) strand	3,909
CDS from (-) strand	3,844
Max. CDS length	32,543 bp
Mean CDS length	1,088 bp
Hypothetical proteins	2,800
Hypothetical proteins %	36.11
tRNA	79
Genes with Pfam domains	5,409 (69.77%)
Genes with COG domains	5,650 (72.88%)
Genes with TIGR domains	3,686 (47.54%)

In *M*. *hansupus* the replication origin was identified at 8,613,829–8,614,077 bp and the corresponding *dnaA* gene was located downstream of the replication origin at 8,646,592–8,645,240 bp. It shows maximum similarity with *M*. *fulvus* replication origin (ORI94030396, 365 bp) with an E-value of 9e-68, 89% identity, and 68% coverage and shows 85% sequence identity with 76% length coverage and an E-value of 1e-29 with *M*. *xanthus DK1622* (ORI92210206, 247 bp).

Putative methylome of the *M*. *hansupus* was identified which revealed m6A based methylation in motifs CCAAGGC (82.4% motifs), CTACNNNNNNTGG (79.2% motifs), CCANNNNNNGTAG (78.1% motifs), SCCCGCA (53.3% motifs), WCCCGCAWG (45.2% motifs) and GATC (31.9% motifs) at 4th, 3rd, 3rd, 7th, 7th and 2nd positions respectively. We identified type I methylases (specific to Adenine) involved in Type I R&M system (AKQ64130, AKQ67990, AKQ68170 and AKQ68203; having N6_Mtase (PF02384)) but Type II methylases corresponding to Type II R&M systems could not be identified. We also found m4C methylation in motif GCGSYDTY (in only 8.3% motifs) at C2 position. We could not identify corresponding N4-methylcytosine (m4C) methylase while other methylases having Pfam domain N6_N4_Mtase (PF01555), which function as both N-4 cytosine-specific and N-6 Adenine-specific DNA methylases, were identified in *M*. *hansupus* genome (AKQ64825, AKQ65130, AKQ65131, AKQ66512 and AKQ67727). These findings are in accordance with the REBASE database of DNA restriction and modification enzymes [40].

### Genomic overview of the *Myxococcus* clade

At the time of this study, five genomes were available in the genera *Myxococcus viz*., *M*. *xanthus* DK1622 [25] (*MxDK1622*), *M*. *fulvus* HW-1 [26] (*Mf*), *M*. *stipitatus* [16] (*Ms*), *M*. *xanthus* DZ2 [18] (*MxDZ2*) and *M*. *xanthus* DZF1 [17] (*MxDZF1*) (Table 2). Among the *M*. *xanthus* strains, *MxDZ2* is known to be the parent strain of both *MxDK1622* and *MxDZF1* [41]. Including *M*. *hansupus*, these genomes represent the *Myxococcus* clade belonging to family Cystobacteraceae under suborder Cystobacterineae of the order *Myxococcales*. Among these *MxDZF1* and *MxDZ2* are draft assemblies with 75 and 87 contigs respectively. Noticeably, genome size of non-Myxococcales *Deltaproteobacteria* members varies in the range of 2 to 7 Mbp which is relatively smaller as compared to the *Myxococcus* genomes which vary between 9 Mbp to 10.35 Mbp (Table 2). Such genome expansion has been attributed to gene duplication, gene rearrangement, and horizontal gene transfer events [19, 42]. All these strains are reported to undergo developmental program leading to fruiting body formation and can perform gliding motility. Owing to such atypical characteristics, these bacteria pursue a complex life cycle that requires a wide range of proteins functioning coherently. The increased protein content in order Myxococcales ranging from 7400–8200 as compared to 4000–5000 in non-Myxococcales δ-proteobacteria is perhaps in part involved in regulatory functions as reported in earlier studies [15, 25].

**Table 2:** General features of genus *Myxococcus* genomes as annotated by RAST.

Organism name	*M*. *fulvus* HW-1	*M*. *stipitatus* DSM 14675	*M*. *xanthus* DK 1622	*M*. *hansupus*	*M*. *xanthus* DZF1	*M*. *xanthus* DZ2
Bio Project Number	PRJNA68443	PRJNA186549	PRJNA58003	PRJNA167109	PRJNA199916	PRJNA199464
NCBI Accession	NC_015711.1	NC_020126.1	NC_008095.1	CP012109	AOBT00000000	AKYI00000000
Chromosome/Contigs	1	1	1	1	75	87
GC %	70.6	69.2	68.9	69.2	68.8	68.9
Size (Mbp)	9	10.35	9.14	9.49	9.28	9.27
Genes [RAST]	7,433	8,293	7,524	7,753	7,700	7,689
Coding% [RAST]	89.27	90.22	89.52	88.87	89.12	88.90
Hypothetical proteins (%)	3,002 (40.39)	3,567 (43.01)	2,942 (39.10)	2,800 (36.11)	3,092 (40.16)	3,266 (42.46)
tRNA [RAST]	76	85	75	79	61	61

### Comparison of annotation pipelines

Various optimized genome annotation pipelines such as RAST [22], Glimmer [29], xBASE [30], PGAAP, JCVI, IGS, and IMG-ER have been used to predict and annotate genes. As the genomes compared here have been annotated using different annotation pipelines at different time points by various groups, therefore we have reannotated the genomes in order to have a consistent and updated annotation of all genomes [43]. Comparative annotation studies were performed to map the annotations with each other to ensure that none of the coding regions in genomes is missed out due to algorithm limitations. We have used the annotation pipelines of RAST, xBASE and Glimmer and also compared these annotations with the original datasets available at NCBI. Comparative mapping studies of all datasets illustrate that annotations using RAST server, GLIMMER, xBASE and original dataset (from NCBI) are comparable to each other with ~97% of proteins being shared amongst them (data not shown).

**Fig 3:**
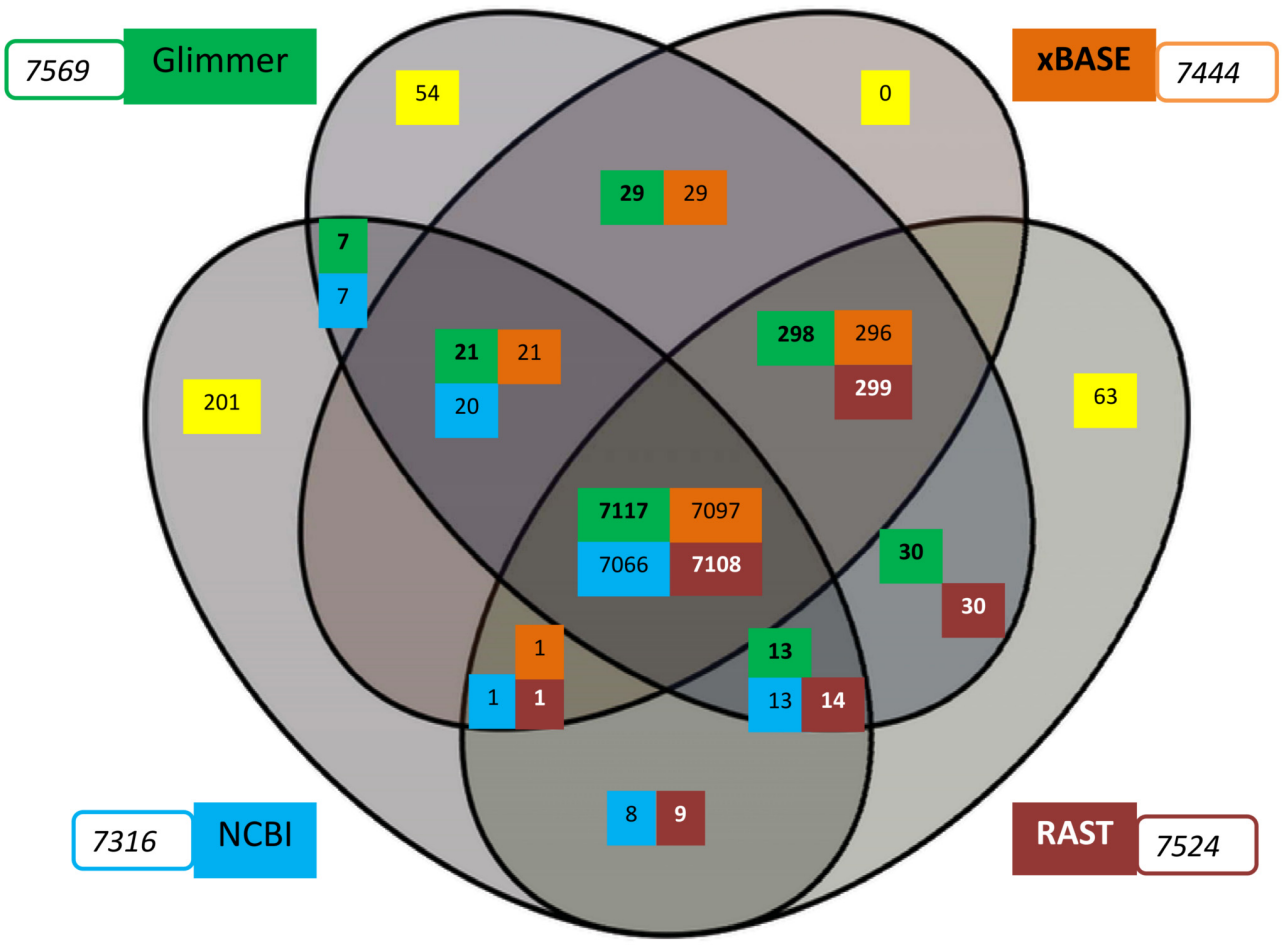
Venn diagram of comparative annotations of *Myxococcus xanthus* DK1622 using RAST, Glimmer, xBASE and the original NCBI annotation. All genome annotations were mapped to each other using BLASTp [E-value cutoff: 1e^-5^]. The diagram depicts the homologous proteins shared between two or more annotations (overlapping area) along with unique proteins (yellow shade). RAST, Glimmer, xBASE and the original NCBI annotations are shown in brick red, green, orange and blue colors respectively. The number of annotated proteins using the respective annotation pipeline is shown in the box.

Taking *Mx*DK1622 genome as a model, we analyzed the results from different pipelines in order to compare the robustness of the annotation statistics (Fig 3). It was observed that annotations from different pipelines are comparable owing to the similar distribution of proteins, albeit several unique proteins are predicted from different pipelines. This exercise suggests that some gene(s) could get overlooked when using a single annotation protocol. Therefore, multiple annotations i.e., RAST, GLIMMER, and xBASE were used for comparative studies, whereas RAST annotations were used for genome-based studies for uniformity.

### Phylogenetic analysis of the *Myxococcus* clade

In genus *Myxococcus*, more than eight species had been reported many of which have been taxonomically reclassified in the absence of respective type strains [44, 45]. Presently this genus consists of *M*. *xanthus*, *M*. *virescens*, *M*. *flavescens*, *M*. *stipitatus* and *M*. *macrosporus*, which differ in the morphology of their vegetative cells and fruiting body, along with pigment formation during swarm growth [45]. All species exhibit typical long and rod-shaped morphology during vegetative states with varied cell sizes [45]. During fruiting body formation, these bacteria display diverse and distinct morphology [20, 46]. Given their close relationship and overlapping morphological features, the taxonomic placement of *Myxococcus* strains is difficult. For instance, in literature *M*. *macrosporus* has been referred to as *Corallococcus macrosporus* but is regarded as a species of the *Myxococcus* genera [47]. Here we discuss the taxonomic position of *M*. *hansupus* based on 16S rRNA, housekeeping genes, and genome-genome distance based phylogeny. The resulting tree from 16S rRNA sequences was not able to resolve all species of the *Myxococcus* genus (S1 Fig). The sequence similarities within all *Myxococcus* spp. 16S rRNA sequences were more than 96%. *Mh* shows maximum similarity with *Mf* (99.45%) followed by *Mx* (99.24%) and then *Ms* (98.28%) suggesting its close relationship to *Mf*. The tree correctly grouped *M*. *xanthus* strains i.e. *MxDK1622*, *MxDZ2* and *MxDZF1* strains, confirming their closeness with each other. Some irregularities in the taxonomic tree include the positions of *M*. *flavescens* NBRC 100081 and *M*. *flavescens* NBRC100077 similar to what has been reported previously [45].

**Fig 4:**
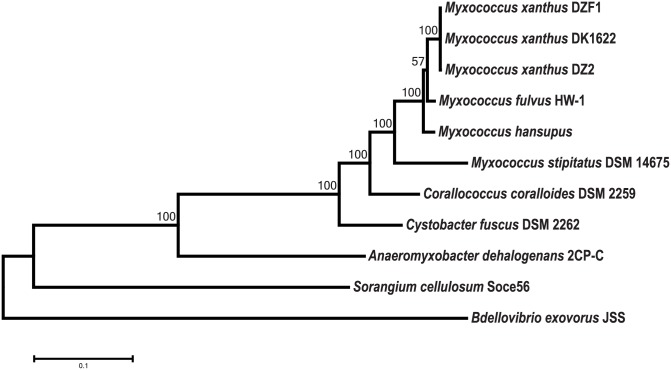
Phylogeny based on housekeeping proteins. Twenty-eight concatenated housekeeping proteins were used to generate ML based phylogenetic tree using MEGA 6.06 [model: JTT matrix; bootstrap: 100]. *Corallococcus coralloides* DSM 2259, *Cystobacter fuscus* DSM 2262, *Anaeromyxobacter dehalogenans* 2CP-C, *Sorangium cellulosum* Soce56, and *Bdellovibrio exovorus* JSS were used as outgroup species in this study. Bootstrap values corresponding to the tree nodes are provided.

In spite of its popularity, 16S rRNA is not a credible marker for taxonomic placement below the genus level [48], therefore housekeeping gene analysis was performed to validate the taxonomic relationship among *Myxococcus* genus. The phylogenetic analysis of 28 housekeeping genes [34] (Fig 4) of the *Myxococcus* clade and five outgroups, reveals a similar tree topology as obtained using 16S rRNA and supports the assertion that *Mh* is closely related to *Mf* followed by other *Mx* species. *MxDK1622*, *MxDZ2*, and *MxDZF1* were placed together, similar to the 16S rRNA based tree. We have also estimated DNA-DNA hybridization (DDH) values between genus *Myxococcus* members using GGDC (Genome-To-Genome Distance Calculator) server [49] which uses GBDP strategy (Genome Blast Distance Phylogeny) (S1 Table). *Mh* genome shares lowest intergenomic distance (highest DDH value) with *Mf* genome. The maximum DDH value of 62.1% with *Mf* further suggests that *Mh* is a novel species within the genus *Myxococcus* as for two organisms to belong to the same species, DDH value should be greater than 70% [49].

### Pan Proteome analysis: Core, Dispensable, and Unique proteome

The Pan Proteome is defined as the sum total of protein content associated with more than two species; and consists of the Core Proteome, Dispensable Proteome and Unique Proteome [50, 51]. The total proteome of six *Myxococcus* genomes consists of 46,392 proteins with 7,901 orthologous protein clusters (S2 Table), where a cluster signifies one representative from each genome. The percentages of proteins from different members in the clusters are *Mf*: 82.09%, *Ms*: 66.44%, *MxDK1622*: 94.90%, *Mh*: 81.39%, *MxDZF1*: 94.27% and *MxDZ2*: 94.33%. Among these, 4,693 clusters are found to be conserved in all the genomes and define the core proteome for the *Myxococcus* clade. The core proteome accounts for 56.6–63% of the total protein content in each genome and consist of genes that are responsible for essential biological functions such as homeostasis, housekeeping functions and maintaining morphological, developmental and physiological features of the organism. The function profile analysis of the core proteome depicts that 5.4% of the proteins are involved in signal transduction while 45% of the core proteome is involved in housekeeping functions such as cell wall/membrane biogenesis (M), amino acid transport (E), translation and ribosome biogenesis (J), post-translational modifications (O), energy production (C), lipid transport (I), replication (L), carbohydrate transport (G), secondary metabolism biosynthesis (Q) and cell motility (N). Fifteen percent of the proteins were assigned to COG’s general function (R) category while the remaining 35% could not be attributed to any known function. The proteins sharing orthology within two or more genomes, but not in all genomes under study, are defined as the dispensable proteome. The dispensable proteome varies from 9.85% (in *Ms*) to 33% (*Mx* strains) among the genomes, a majority of which is likely involved in species-specific functions. The dispensable proteome consists of genes that allow the organism to sustain its species level diversity and participate in the regulation of accessory functions [50]. The analysis reveals that *M*. *stipitatus* show the minimum orthology protein pairs with other species followed by *Mh* and *Mf*.

**Fig 5:**
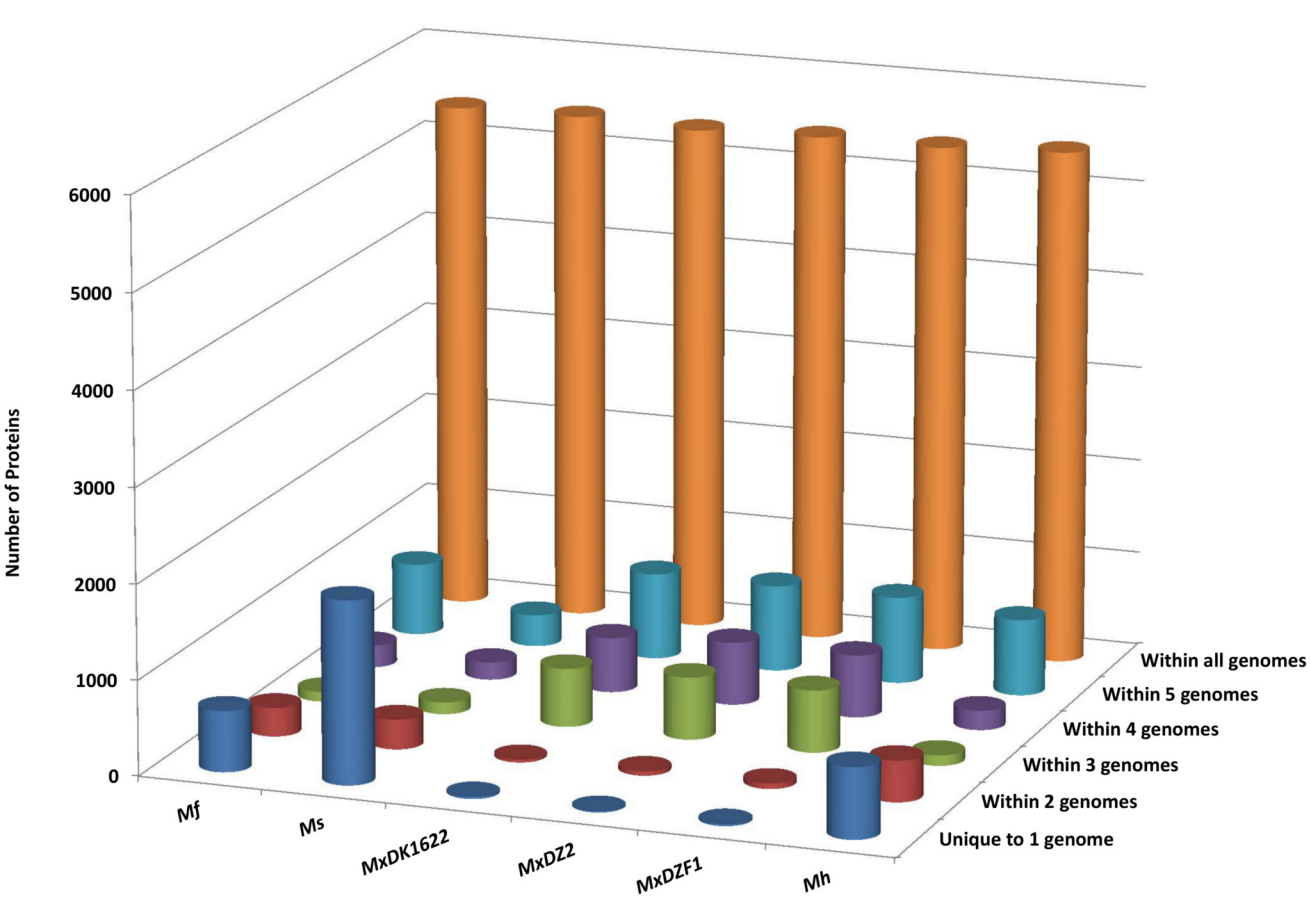
Comparative representation of homologous protein distribution within the genomes. Proteome dataset from each genome was subjected to BLASTp to identify homologous proteins between the genomes with an E-value cutoff of 1e-5, query coverage of 50% and identity of 35%. Protein distribution between different combinations of genomes was identified and is represented as a 3D-graph. X, Y and Z-axis respectively denote genome name, the number of proteins and the genome combination.

Homology studies among the genomes provide insights into the extent of duplicated genes, thereby explaining an important factor of genome expansion. Homologous genes among all the genomes and unique genes in each of the genome were identified in this study. There are 46,392 proteins encoded by all six genomes out of which 32,415 proteins (69.87% of total proteins) have homologs in all genomes, which accounts for 5,453 proteins in *Mh*; 5,395 in *MxDZF1*; 5,401 in *MxDZ2*; 5,367 in *MxDK1622*; 5,406 in *Ms* and 5,393 proteins in *Mf*; representing 70.33, 70.06, 70.24, 71.33, 65.19 and 72.55% of proteins from each genome (Fig 5). The remaining proteins are either restricted to a single genome or present in two or more genomes (Table 3). An all-to-all protein content comparison matrix reveals that *Mh* shares 82.7% genes of *Mf* and 83.36% of *MxDK1622* while only 76.82% genes of *Ms* are mapped to *Mh* (S3 Table). Likewise, *Mf*, *MxDK1622*, and *Ms* share 85.48%, 84.77% and 71.92% genes of *Mh* respectively. *MxDK1622*, *MxDZ2*, and *MxDZF1* are quite similar to each other, with only 0.5–1.0% difference in their protein content. This suggests that the genomes herein share ~80% of their protein content while diversity and uniqueness in each genome are achieved by the remaining 20% of the genes. Complete chromosomes of *M*. *hansupus*, *M*. *fulvus* HW-1, *M*. *stipitatus* DSM 14675 and *M*. *xanthus* DK1622 were aligned with each other and syntenic plots for all combinations of genomes were generated (S2 Fig). Blue and red dots represent putative homologous regions in positive and negative DNA direction between two genomes as identified by sequence similarity. These plots revealed large identical syntenic blocks suggesting relative closeness between the genus *Myxococcus* genomes. We also identified various insertions and translocations within these genomes.

**Table 3:** A binary map depicting cluster of homologous proteins among all genomes.

*M*. *fulvus*	*M*. *stipitatus*	*M*. *xanthus* DK1622	*M*. *xanthus* DZ2	*M*. *xanthus* DZF1	*M*. *hansupus*	Presence in genomes	Total proteins	Total proteins%
a	a	a	a	a	P	1	747	1.61
a	a	a	a	P	a	1	18	0.04
a	a	a	P	a	a	1	12	0.03
a	a	P	a	a	a	1	19	0.04
a	P	a	a	a	a	1	1929	4.16
P	a	a	a	a	a	1	643	1.39
a	a	a	a	P	P	2	3	0.01
a	a	P	a	a	P	2	5	0.01
a	P	a	a	a	P	2	430	0.93
P	a	a	a	a	P	2	453	0.98
a	a	a	P	P	a	2	77	0.17
a	a	P	a	P	a	2	30	0.07
P	a	a	a	P	a	2	1	0.00
a	a	P	P	a	a	2	16	0.03
a	P	a	P	a	a	2	1	0.00
P	a	a	P	a	a	2	1	0.00
a	P	P	a	a	a	2	2	0.00
P	a	P	a	a	a	2	4	0.01
P	P	a	a	a	a	2	157	0.34
a	a	a	P	P	P	3	8	0.02
a	a	P	a	P	P	3	1	0.00
P	a	a	a	P	P	3	4	0.01
a	a	P	P	a	P	3	5	0.01
a	P	P	a	a	P	3	8	0.02
P	a	P	a	a	P	3	3	0.01
P	P	a	a	a	P	3	295	0.64
a	a	P	P	P	a	3	1869	4.03
a	P	a	P	P	a	3	18	0.04
P	a	a	P	P	a	3	18	0.04
a	P	P	a	P	a	3	4	0.01
P	a	P	a	P	a	3	8	0.02
a	P	P	P	a	a	3	12	0.03
P	a	P	P	a	a	3	9	0.02
P	P	P	a	a	a	3	4	0.01
a	a	P	P	P	P	4	830	1.79
a	P	a	P	P	P	4	1	0.00
P	a	a	P	P	P	4	17	0.04
a	P	P	a	P	P	4	2	0.00
P	a	P	a	P	P	4	8	0.02
P	P	a	a	P	P	4	2	0.00
a	P	P	P	a	P	4	6	0.01
P	a	P	P	a	P	4	1	0.00
P	P	a	P	a	P	4	4	0.01
P	P	P	a	a	P	4	11	0.02
a	P	P	P	P	a	4	653	1.41
P	a	P	P	P	a	4	976	2.10
P	P	a	P	P	a	4	1	0.00
P	P	P	P	a	a	4	15	0.03
a	P	P	P	P	P	5	870	1.88
P	a	P	P	P	P	5	3148	6.79
P	P	a	P	P	P	5	10	0.02
P	P	P	a	P	P	5	24	0.05
P	P	P	P	a	P	5	13	0.03
P	P	P	P	P	a	5	571	1.23
P	P	P	P	P	P	6	32415	69.87

The map depicts the count of homologous proteins along with their presence [denoted as ‘P’] and absence [denoted as ‘a’] in the genome combinations. The numbers in the last column represent the fraction of the proteins from all genomes. Proteins present in various combinations such as one (unique), two, three, four, five and all genomes are shown.

Unique proteins were also identified using BLAST analysis. These proteins are present only in one genome with no homologs in other genomes. The number of unique proteins varies from 12 to 1,929 in genus *Myxococcus* members (Fig 5). This account for 0.16% unique proteins in *MxDZ2*, 0.23% in *MxDZF1*, 0.25% in *MxDK1622*, 8.65% in *Mf*, 9.63% in *Mh* and 23.26% in *Ms*. The large number of unique proteins, mostly annotated as hypothetical proteins with unknown functions, is suggestive of high genomic diversity within the same genus.

### Protein clustering

We have clustered each proteome dataset, to compare the homologous proteins within each genome. Our clustering analysis suggests that these six genomes have 590–660 protein clusters sharing on an average of 2424 proteins in each genome which may be represented as multi-copy or duplicated proteins (S4 Table). Each cluster contains between 2 to 431 proteins. The remaining, 5308 proteins on average are singletons and have no homologs within the genome. Our analysis suggests that on average 31.33% of the proteins in each genome are present in multiple copies in the *Myxococcus* genomes. Among these duplicated proteins the maximum representation was from response regulators, protein kinases, ABC transporters, long-chain fatty acid CoA ligase, short-chain dehydrogenase and LysR family transcriptional regulator proteins.

We also performed Pfam domain and clan-based clustering for the six *Myxococcus* proteomes along with rest of the Myxobacteria (*Sorangium*, *Cystobacter*, *Chondromyces*, *Plesiocystis*, *Stigmatella*, *Corallococcus*, *Haliangium*, *and Anaeromyxobacter*) and representative proteomes of non-Myxococcales δ-proteobacteria (S5 Table). We found that several Pfam clans such as protein kinase domain [CL0016], PP-binding (CL0314), PKinase (CL0016), CoA-acyltrans (CL0149), Peptidase_PA (CL0124), GroES (CL0296), AB_hydrolase (CL0028), Thiolase (CL0046), CheY (CL0304), AMP-binding_C (CL0531), HTH (CL0123) etc. are overrepresented in order Myxococcales members by more than 200% as compared to non-Myxococcales δ-proteobacteria. Besides this, many Pfam clans such as EGF (CL0001), Trefoil (CL0066), gCrystallin (CL0333), Aerolisin_ETX (CL0345), Hydrophilin (CL0385), Viral_Gag (CL0148), zf-FYVE-PHD (CL0390), HMG-box (CL0114), PLAT (CL0321), EsxAB (CL0352), Frag1-like (CL0412), Hexosaminidase (CL0546) etc. are particularly present in genus *Myxococcus* members and not in the non-Myxococcales δ-proteobacteria. The presence of overrepresented and unique Pfam families in *Myxococcus* genomes as compared to other non-Myxococcales δ-proteobacterial genomes is suggestive of the nature of genome expansion and could probably help these organisms to adapt to diverse habitats and in leading a complex life cycle. Such adaptability could have been achieved by gain, loss or duplication of gene/protein content [19, 52]. Our results are in accordance with reports that attribute gene duplication as one of the main driving force behind genome expansion in *Myxococcus* genomes [42].

## Conclusion

The current study reports the complete 9.5 Mbp genome of a novel Myxobacteria, *M*. *hansupus* and its comparative analysis with five previously available *Myxococcus* genomes. 16S rRNA, housekeeping genes phylogeny, and genome-genome distance suggest this organism is a novel species of the genus *Myxococcus*. Overall protein similarity among six *Myxococcus* genomes, which include four different species and three strains of *M*. *xanthus*, help define the core, dispensable and unique proteomes for genus *Myxococcus*. Orthology analysis revealed ~60% of the proteins as the core proteome whereas homology studies identified the presence of ~70% of the total proteome in these closely related genus *Myxococcus* members. The wide genome diversity at species level within genus *Myxococcus* is revealed by the presence of large number of unique proteins, e.g. as high as 1,929 unique proteins in *M*. *stipitatus* genome. Protein sequence clustering reveals that 31% of the total protein content is present in multiple copies with a majority of the proteins functioning as response regulators, kinases and ABC transporters. The presence of several overrepresented Pfam clans and their constituting families helps in identifying the genome expansion in *Myxococcus* genomes as compared to other non-Myxococcales δ-proteobacteria genomes.

## Supporting Information

S1 Fig Phylogenetic analysis of genus *Myxococcus* 16S rRNA. MEGA 6.06 was used to generate a maximum likelihood tree [model: Tamura 3-param; bootstrap: 1000]. Different leaf colors were used in the tree to demarcate species; *M*. *xanthus*: navy blue, *M*. *fulvus*: dark teal, *M*. *stipitatus*: yellow, *M*. *virescens*: green, *M*. *macrosporus*: dark brown and *M*. *flavescens*: light brown. Black circle represents complete genomes and red semicircle represents the draft genomes. Bootstrap values are provided corresponding to the tree nodes. *Corallococcus coralloides*, *Cystobacter fuscus*, *Anaeromyxobacter dehalogenans*, *Sorangium cellulosum*, and *Bdellovibrio exovorus* were used as outgroup species in this study.
(EPS)

S2 Fig Syntenic dot plot between genus *Myxococcus* complete genomes. Complete genomes of *M*. *hansupus*, *M*. *fulvus* HW-1, *M*. *stipitatus* DSM 14675 and *M*. *xanthus* DK1622 were used in this study. Blue and red dots represent putative homologous regions between two genomes in the positive and negative directions respectively as identified by sequence similarity. Panel A, B and C represent the dot plots for *M*. *hansupus* aligned against *M*. *xanthus*, *M*. *fulvus* and *M*. *stipitatus* respectively. Panel D and E represent the dot plots for *M*. *fulvus* aligned against *M*. *stipitatus* and *M*. *xanthus*. Panel F represents the dot plot analysis of *M*. *xanthus* aligned against *M*. *stipitatus*.
(EPS)

S1 Table Genome-to-genome distance between *M*. *hansupus* and its neighbors. The genome-to-genome distance between *M*. *hansupus* and other myxobacterial genomes (*Corallococcus coralloides* DSM 2259, *Cystobacter fuscus* DSM 2262, *Anaeromyxobacter dehalogenans* 2CP-1, *and Sorangium cellulosum* Soce56) and *Bdellovibrio bacteriovorus* W using GGDC (version 2.0 and Formula 2) is shown here. Red-yellow-green shading depicts decreasing closeness based on DDH values.
(XLS)

S2 Table Matrix of orthologous protein sets for six *Myxococcus* genomes. The matrix depicts the count of orthologous proteins [column J] along with their presence [denoted as P in white shade] and absence [shaded black] in all possible genome combinations [column I]. Orthologous proteins present in two, three, four, five and all genomes are shaded gray, light green, dark purple, blue and dark green respectively.
(XLS)

S3 Table All-to-all protein content comparison matrix. All proteins were mapped between two genomes and their mapping percentage to each genome is represented here with high to low (red-yellow-green) shading order. The matrix should be read as % proteins of [row] genome mapped against [column] genome.
(XLS)

S4 Table Protein clustering analysis between *Myxococcus* genomes. BLASTp results were filtered on the basis of cut-off values [E-value: 1e^-5^, query coverage: 50% and identity: 35%] and protein homologs were clustered. Singleton proteins, proteins present in clusters and numbers of clusters are shown in column D, E and G.
(XLS)

S5 Table Pfam clans based clustering in six *Myxococcus* genomes and comparative distribution with rest of the myxobacteria and non-Myxococcales δ-proteobacterial members. Apart from six *Myxococcus* genomes [column C-H], rest of the myxobacteria [column K] include *Sorangium*, *Cystobacter*, *Chondromyces*, *Plesiocystis*, *Stigmatella*, *Corallococcus*, *Haliangium*, *and Anaeromyxobacter*. Non-Myxococcales δ-proteobacteria [column L] include *Bacteriovorax marinus* SJ, *Bdellovibrio bacteriovorus* HD100, *Bilophila wadsworthia*, *Deferrisoma camini*, *Desulfarculus baarsii* DSM 2075, *Desulfatibacillum alkenivorans* AK 01, *Desulfatiglans anilini*, *Desulfatirhabdium butyrativorans*, *Desulfobacca acetoxidans* DSM 11109, *Desulfobacter curvatus*, *Desulfobacterium autotrophicum* HRM2, *Desulfobacula toluolica* Tol2, Desulfobulbaceae bacterium BRH c16a, *Desulfobulbus propionicus* DSM 2032, *Desulfocapsa sulfexigens* DSM 10523, *Desulfococcus oleovorans* Hxd3, *Desulfocurvus vexinensis*, *Desulfohalobium retbaense* DSM 5692, *Desulfomicrobium baculatum* DSM 4028, *Desulfomonile tiedjei* DSM 6799, *Desulfonatronum thioautotrophicum*, *Desulforegula conservatrix*, *Desulfotalea psychrophila* LSv54, *Desulfotignum balticum*, *Desulfovermiculus halophilus*, *Desulfovibrio hydrothermalis* AM13, *Desulfurella acetivorans*, *Desulfurivibrio alkaliphilus* AHT2, *Desulfuromonas acetoxidans*, *Geoalkalibacter ferrihydriticus*, *Geobacter sulfurreducens* KN400, *Geopsychrobacter electrodiphilus*, *Hippea maritima* DSM 10411, *Lawsonia intracellularis* N343, *Pelobacter carbinolicus* DSM 2380, *Syntrophobacter fumaroxidans* MPOB, *Syntrophorhabdus aromaticivorans* and *Syntrophus aciditrophicus* SB. Pfam clan number and clan name are shown in column A and B. The numbers of proteins per clan in six *Myxococcus* genomes are represented [column C-H] with high to low (red-yellow-green) shading order. A similar shading is used for the numbers of proteins per clan in average *Myxococcus* [column J], average rest-Myxobacteria [column K] and average-non-Myxococcales δ-proteobacteria [column L]. In column M, % increase/decrease of numbers of proteins per clan in *Myxococcus* is depicted as compared to δ-proteobacteria.
(XLS)
